# Global hypomyelination of the brain white and gray matter in schizophrenia: quantitative imaging using macromolecular proton fraction

**DOI:** 10.1038/s41398-021-01475-8

**Published:** 2021-06-30

**Authors:** Liudmila P. Smirnova, Vasily L. Yarnykh, Daria A. Parshukova, Elena G. Kornetova, Arkadiy V. Semke, Anna V. Usova, Anna O. Pishchelko, Marina Y. Khodanovich, Svetlana A. Ivanova

**Affiliations:** 1 Laboratory of Molecular Genetics and Biochemistry, Mental Health Research Institute, Tomsk National Research Medical Center of the Russian Academy of Sciences, Tomsk, 634014 Russian Federation; 2 Department of Radiology, University of Washington, Seattle, WA 98109 USA; 3 Laboratory of Neurobiology, Research Institute of Biology and Biophysics, Tomsk State University, 634050 Tomsk, Russian Federation; 4 Department of diagnostic radiology Cancer Research Institute, Tomsk National Research Medical Center of the Russian Academy of Sciences, Tomsk, 634050 Russian Federation

**Keywords:** Pathogenesis, Schizophrenia, Molecular neuroscience

## Abstract

Myelin deficiency is commonly recognized as an important pathological feature of brain tissues in schizophrenia (SZ). In this pilot study, global myelin content abnormalities in white matter (WM) and gray matter (GM) of SZ patients were non-invasively investigated using a novel clinically-targeted quantitative myelin imaging technique, fast macromolecular proton fraction (MPF) mapping. MPF maps were obtained from 23 healthy subjects and 31 SZ patients using a clinical 1.5T magnetic resonance imaging (MRI) scanner. Mean MPF in WM and GM was compared between the healthy control subjects and SZ patients with positive and negative leading symptoms using the multivariate analysis of covariance. The SZ patients had significantly reduced MPF in GM (*p* < 0.001) and WM (*p* = 0.02) with the corresponding relative decrease of 5% and 3%, respectively. The effect sizes for the myelin content loss in SZ relative to the control group were 1.0 and 1.5 for WM and GM, respectively. The SZ patients with leading negative symptoms had significantly lower MPF in GM (*p* < 0.001) and WM (*p* = 0.003) as compared to the controls and showed a significant MPF decrease in WM (*p* = 0.03) relative to the patients with leading positive symptoms. MPF in WM significantly negatively correlated with the disease duration in SZ patients (Pearson’s *r* = −0.51; *p* = 0.004). This study demonstrates that chronic SZ is characterized by global microscopic brain hypomyelination of both WM and GM, which is associated with the disease duration and negative symptoms. Myelin deficiency in SZ can be detected and quantified by the fast MPF mapping method.

## Introduction

Myelin abnormalities in schizophrenia (SZ) have been attracting substantial interest over the past two decades^1–7^. A number of post-mortem investigations of the SZ brain confirmed various aspects of myelin disorganization and dysfunction associated with this disease. Electron microscopy studies demonstrated abnormalities in the myelin sheath ultrastructure associated with degeneration of oligodendroglia in both gray matter (GM) and white matter (WM) of SZ patients^8–10^. Post-mortem genetic transcriptome studies (detailed review can be found in ref. ^11^) identified downregulation of a series of genes encoding key proteins related to the oligodendrocyte function and myelin biosyntheses, such as myelin basic protein (MBP), myelin oligodendrocyte glycoprotein, and transferrin^12,13^ in various anatomic regions of the brain. Histological studies reported a reduced myelin density in the cortex according to MBP immunostaining^14^ and luxol-fast-blue staining^15^, as well as a decreased amount of oligodendrocytes in the hippocampus^16,17^, cortical GM^18,19^, and subcortical WM^19^. Impaired production of myelinating oligodendrocytes from oligodendrocyte precursor cells also has been found in SZ^20^. While the etiology of oligodendroglial dysfunction and related dysmyelination in SZ is still poorly understood, it is commonly recognized to date that oligodendrocyte and myelin damage represents a fundamental aspect of the molecular and cellular brain pathology in this disease and may become a target for future therapeutic interventions^6,7^.

Subtle structural and microstructural brain abnormalities in SZ were detected in numerous non-invasive magnetic resonance imaging (MRI) studies. A number of morphological studies demonstrated regional signs of accelerated GM and WM atrophy as compared to the normal population and aberrant cortical gyrification^21,22^. Certain microstructural features of brain tissues in SZ reported in the literature appeared consistent with abnormal myelination. The vast majority of such findings have been based on diffusion tensor imaging (DTI), which produces a series of indices reflecting the integrity of WM fiber tracts^23,24^. The common observations in SZ typically linked to hypomyelination include reduced fractional anisotropy^23–26^ and increased radial diffusivity^25,26^. However, these DTI parameters strongly depend on the diameter, density, and spatial coherence of axons^27,28^, and, therefore, cannot be used as myelin biomarkers in the presence of a diverse axonal population. Another widely used SZ research technique is magnetization transfer (MT) imaging, which produces a semi-quantitative index, MT ratio (MTR) that has been commonly interpreted as a marker of brain tissue myelination. A number of studies (reviewed in^29^) found a reduced MTR in SZ, though some reports indicated no effect or an increase in this parameter^30–32^. However, MTR was shown to have low specificity and sensitivity to myelin because of its dependence on both cross-relaxation and longitudinal relaxation in tissues^33,34^. More specific myelin imaging measures, such as myelin water fraction (MWF) and T1/T2 signal ratio were applied in a few studies of SZ^35–39^. MWF demonstrated regional reductions in WM of SZ patients relative to the normal control population^35,36^ but did not show changes in the first-episode SZ^37^. T1/T2 signal ratio showed a global decrease in WM and GM in SZ in one study^38^ and opposite regional trends of either decrease or increase in different cortical areas of the first-episode SZ patients in another study^39^. It also should be pointed out that both MWF and T1/T2 signal ratio relies on magnetic relaxation as the source of quantitative information, and, therefore, are influenced by any factors affecting T1 and T2 relaxation times, such as concentrations of paramagnetic ions including iron^40,41^. In brain diseases, alterations of the iron content are a common cause of T1 and T2 changes^42^, and the corresponding confounding effect cannot be ignored in the applications for the above techniques. In summary, a general limitation of the previous neuroimaging studies of myelin abnormalities in SZ is the use of imaging biomarkers, which do not provide sufficient sensitivity and specificity to enable unambiguous interpretation of the results in terms of the myelin content changes. A more accurate and precise myelin imaging technique is required for the prospective use as both a measure of the disease progression and a potential surrogate marker of the effect of therapies targeted at the treatment of oligodendroglial dysfunction.

Several more specific myelin imaging techniques based on the MT effect, such as relaxation-independent MT saturation^43^, inhomogeneous MT^44^, and macromolecular proton fraction (MPF)^45,46^ mapping, were recently developed but have not been applied in SZ research. One of these methods, fast MPF mapping^45,46^ yields the maps of the fraction of macromolecular protons involved in cross-relaxation with free water protons. During past years, this technique showed promise as a clinically targeted tool for the assessment of myelin damage and development in the human brain^34,47–51^. MPF has been validated as a myelin biomarker in a number of animal model studies and demonstrated a close linear relationship with the myelin content in both WM and GM^33,52–55^. A recent meta-analysis^55^ indicated high specificity of MPF to myelin and its metrological superiority as compared to other myelin imaging biomarkers. In the pilot clinical studies, MPF mapping enabled the quantitative assessment of microscopic demyelination in normal-appearing WM and GM caused by multiple sclerosis^34,47^ and mild traumatic brain injury^48^. The method also showed the capability to quantify subtle changes in the myelin content at the earliest stages of prenatal brain development^49,50^ and in the course of brain maturation during adolescence^51^. In the recent synthetic-reference design^46^, MPF mapping utilizes only three source images, thus enabling clinically reasonable acquisition time. In the quantitative aspects, MPF mapping provides high reproducibility^51,56^, independence of magnetic field strength^57,58^, and insensitivity to iron deposition^47^, axonal organization^28,33,53^, and changes in the content of non-myelin cellular components of neural tissues, such as glia and neurons^53^. In view of the above technical advantages, it would be reasonable to investigate the capability of MPF mapping to assess myelination abnormalities in SZ.

The objectives of this pilot study were to test the feasibility of the detection and quantitation of myelin deficiency in SZ at the level of global brain WM and GM measurements using the fast clinically-targeted MPF mapping method and explore potential clinical correlations of the myelin content in the SZ population.

## Materials and methods

### Subjects

Thirty-one patients with schizophrenia were recruited to study. The inclusion criteria were the following: the diagnosis of schizophrenia (F20.0, F20.6), in accordance with the International Statistical Classification of Diseases and Related Health Problems, 10th Revision (ICD-10)^59^ and the Structured Clinical Interview for DSM-IV Axis I Disorders (SCID)), age from 18 to 60 years.

The exclusion criteria were the following: the presence of acute and chronic infectious, inflammatory, autoimmune, or neurological diseases, other organic mental disorders, and mental retardation. The sample was formed from outpatients visiting the clinics of the TNIMTs from August till October 2019 with a continuous observation method. At the time of inclusion in the study, patients were receiving anti-relapse therapy. Most of the patients (81%) have taken atypical antipsychotics in the last 6 months. MRI scan of the brain was performed during the period of therapeutic remission. In order to analyze the MPF maps of the brain, taking into account the clinical characteristics of schizophrenia, the study included patients with different leading symptoms and duration of the disease. The patients were stratified into 2 groups: with positive and negative symptoms, using a clinical interview and the Positive and Negative Syndrome Scale (PANSS) for schizophrenia (Table 1).

**Table 1:** Demographic and clinical characteristics of patients with schizophrenia and healthy control subjects.

	Healthy control subjects	Patients with schizophrenia	Positive schizophrenia	Negative schizophrenia
Numbers	23	31	10	21
Male %	38%	52%	56%	48%
Age	38.58 ± 9.55	38.64 ± 8.98	30.33 ± 4.42	42.17 ± 8.17
Age of manifestation		23.12 ± 4.69	20.56 ± 4.33	23.83 ± 4.44
Duration of disease, years	–	15.12 ± 7.26	9.11 ± 3.58	18.04 ± 6.36
CPZ equivalent (mg/day)	–	273.5 ± 292.1	443.3 ± 412.5	215.9 ± 212.4
PANSS positive		15.9 ± 8.13	21.25 ± 6.1	17.71 ± 5.3
PANSS negative		23.21 ± 4.33	18.50 ± 5.5	28.21 ± 3.8
PANSS general		46.33 ± 8.56	42.75 ± 6.4	51.78 ± 9.1
PANSS total		85.45 ± 17.45	82.51 ± 15.5	97.71 ± 19.2

The control group consisted of 23 healthy subjects. These control subjects were mentally and somatically healthy individuals and were recruited from volunteers from Tomsk State University. They did not have significant differences in sex and age with the group of patients with schizophrenia (*p* = 0.98). The excluding criteria for the controls were the presence of acute and chronic infectious, inflammatory, autoimmune, or neurological diseases and organic, including symptomatic, mental disorders. A subgroup of 8 control participants underwent two repeated MRI examinations with the scan-rescan interval from 1 to 7 days to assess the reproducibility of MPF measurements.

All individuals included in the study gave written informed consent. Ethical approval was granted (protocol N 78/1.2019) by the Local Bioethics Committee of the Mental Health Research Institute in accordance with Helsinki ethics committee guidelines. None of the participants were compromised in their capacity/ability to consent; thus, consent from the next-of-kin was not necessary, and it was not recommended by the local ethics committee. The MRI scanning procedure was well tolerated by all participants.

### MRI data acquisition

MRI data were acquired on a 1.5T clinical scanner (Magnetom Essenza; Siemens, Erlangen, Germany). The fast MPF mapping protocol was implemented using a standard manufacturer’s 3D spoiled gradient-echo sequence according to the single-point synthetic reference method^45,46^. The method is based on the reconstruction of MPF maps from three source images with MT, T1, and proton density (PD) contrast weightings by voxel-wise iterative solution of the pulsed MT equation^45^ with a calculated synthetic reference image for data normalization^46^. The protocol implementation was similar in concept to those used in the earlier fast MPF mapping studies with 1.5T MRI equipment^49,50,60^ and utilized the shortest available repetition time (TR) in the MT-weighted sequence. Acquisition parameters for the source images were as follows:

1. MT-weighted: TR = 20 ms, echo time (TE) = 4.76 ms, flip angle (FA) = 8°, scan time 5 min 40 s;
2. T1-weighted: TR = 16 ms, TE = 4.76 ms, FA = 18°, scan time 4 min 32 s;
3. PD-weighted: TR = 16 ms, TE = 4.76 ms, FA = 3°, scan time 4 min 32 s.

For MT-weighting, a standard manufacturer’s off-resonance saturation pulse with Gaussian envelope, offset frequency of 1.5 kHz, effective FA of 500°, and duration of 7.68 ms was applied. All images were obtained in the sagittal plane with a nominal voxel size of 1.25 × 1.25 × 1.25 mm^3^ (matrix 192 × 192 × 160, field-of-view 240 × 240 × 200 mm^3^), 80% resolution in the phase and slice directions (actual voxel size 1.25 × 1.56 × 1.56 mm^3^), partial Fourier transform with the factor of 7/8 in the slice direction, and single signal acquisition. Each scanning session also included a screening T2-weighted sequence and had a total duration of about 20 min.

### Image processing and analysis

MPF maps were reconstructed using custom C++ language software (available at https://www.macromolecularmri.org/) based on the single-point synthetic reference algorithm^45,46^. The two-pool model parameter constraints in the single-point algorithm were set according to the previous implementation for 1.5T data^49^ as follows: cross-relaxation rate constant *R* = 19 s^−1^; T2 of bound macromolecular protons, *T*_2_^B^ = 10 μs; and the product of the relaxation rate R1 and T2 of free water protons, *R*_1_*T*_2_^F^ = 0.055. The last constraint was used to account for direct saturation of water protons based on the Lorentzian lineshape approximation as detailed earlier^45^. No correction of B0 and B1 field inhomogeneity was performed due to the absence of corresponding field mapping sequences on the used MRI scanner. According to the literature^56^, the impact of B0 and B1 field non-uniformity on the accuracy of MPF measurement in segmented brain tissues at 1.5T is expected to be very small with residual relative errors <1%.

Further processing of the 3D MPF maps was carried out according to the earlier described pipeline^34,48^ using FSL software (FMRIB Software Library v. 6.0; Oxford Centre for Functional Magnetic Resonance Imaging of the Brain, University of Oxford, Oxford, England; available at www.fmrib.ox.ac.uk/fsl/). In brief, MPF maps were skull stripped using the brain-extraction tool BET^61^ and then segmented using the automated segmentation tool FAST^62^ into the four tissue classes. The segmented brain tissues included pure WM, pure GM, mixed voxels containing partial volumes from white and gray matter (PVWGM), and mixed voxels containing a partial volume from cerebrospinal fluid (PVCSF). This segmentation technique was specifically designed for the analysis of MPF maps^34,48^ to alleviate the partial volume effects by separating pure (WM and GM) and mixed (PVWGM and PVCSF) tissue classes and thus enable accurate MPF measurements in WM and GM. The segmentation procedure was initialized with the tissue priors of 13%, 6%, 9%, and 1% for WM, GM, PVWGM, and PVCSF, respectively. The PVWGM and PVSCF tissue classes were used to separate MPF measurements in WM and GM from a potential confounding effect of partial volume averaging. The PVCSF tissue was also used to exclude the residual contribution from cerebrospinal fluid after applying the MPF threshold (MPF > 1%) and to correct for brain masking imperfectness since this tissue class typically absorbs residual non-brain voxels. The PVCSF data were not used in subsequent analyses similar to the earlier studies^34,48^. The resulting binary segmentation masks were applied to calculate mean MPF in WM, GM, and PVWGM tissues.

### Statistical analysis

The normality of continuous data distributions was assessed by the Shapiro-Wilk test within each subject group. Parametric analyses were subsequently used since no significant departures from the normal distribution were identified. The gender distribution and mean age were compared between SZ patients and controls using the Pearson *χ*^2^ test and independent-samples Student *t*-test, respectively. Clinical variables were compared between the patients with positive and negative symptoms using the independent-samples *t-*test. To compare mean MPF values in segmented brain tissues between the participant groups, the multivariate analysis of covariance (MANCOVA) was used with age as a covariate (to adjust for age) and either two (patients and controls) or three (patients with negative and positive symptoms and controls) levels of the group factor. Overall MANCOVA significance was assessed using the Wilks Λ-test. Tukey honest significant difference post-hoc pairwise tests were used to adjust for multiple comparisons. Distinctions in MPF between patients and controls were also characterized by the percentage differences and effect sizes (Cohen’s d) calculated as the ratio of the mean difference to the pooled standard deviation. Pearson’s correlation coefficient (r) was used to test relationships between MPF and clinical variables. For significant correlations, partial correlation coefficients with adjustment for age were also calculated. Scan-rescan reproducibility of MPF measurements in a subgroup of healthy controls was assessed using Bland-Altman plots, paired Student *t*-test, and within-subject coefficient of variation (CoV), which were calculated for each segmented brain tissue class. Two-tailed tests were used in all analyses with a significance level of *P* < 0.05. All statistical analyses were carried out in Statistica 12 for Windows software (StatSoft, Tulsa, OK, USA).

## Results

**Fig. 1:**
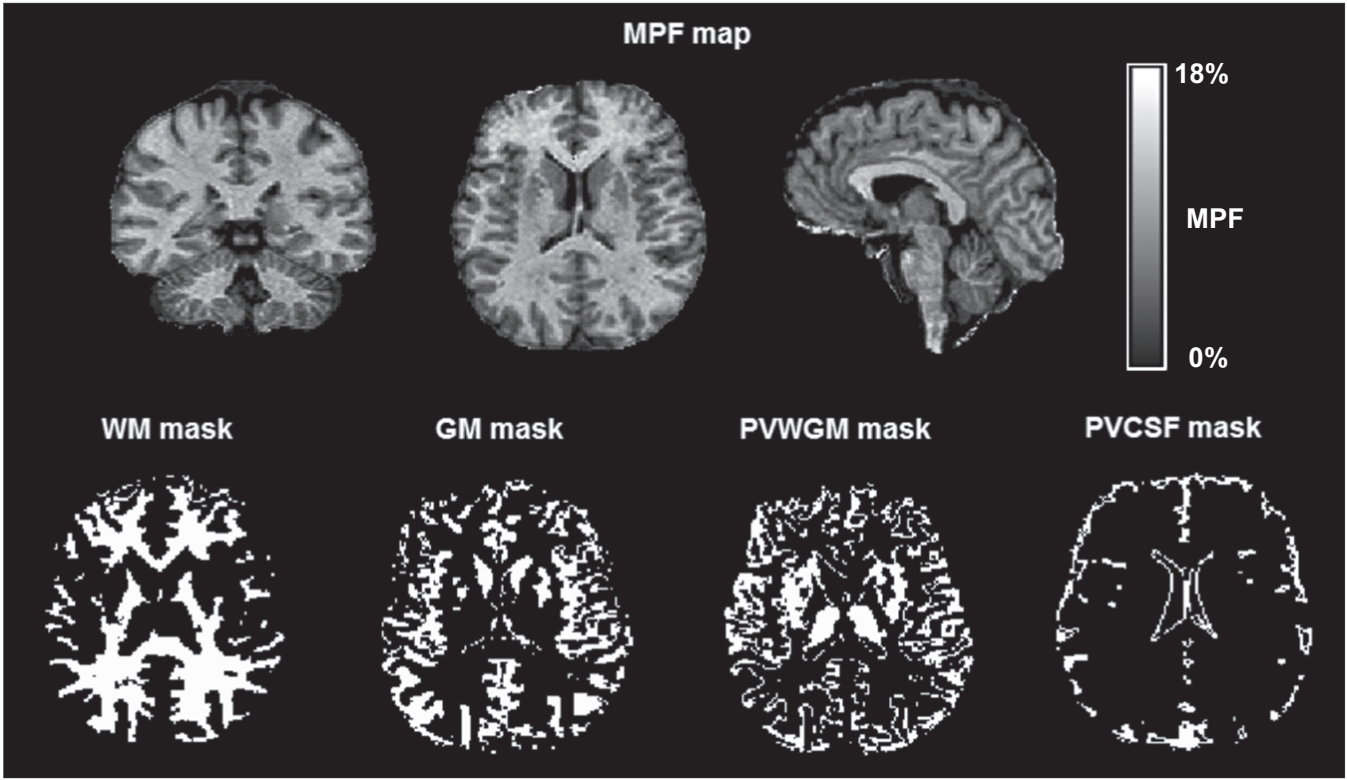
Example three-dimensional MPF macromolecular proton fraction map and binary tissue segmentation masks obtained from a 33-year old male patient with schizophrenia. Segmentation masks correspond to the following tissue classes: white matter (WM), gray matter (GM), and mixed WM and GM (PVWGM).

An example MPF map and the results of brain segmentation for an SZ patient are presented in Fig. 1. Mean MPF values in segmented brain tissues and the results of group comparisons are summarized in Table 2. In comparison between the SZ patients and controls, MANCOVA revealed the significant global effect of the disease (Λ = 0.67, F(3,49) = 7.9, *p* < 0.001) and insignificant influence of the age covariate (Λ = 0.89, F(3,49) = 2.1, *p* = 0.11). The SZ patients had significantly reduced MPF in all brain tissues (Table 2). When the SZ patients with positive and negative symptoms were compared to controls, the group factor remained significant (Λ = 0.60, F(6,96) = 4.7, *p* < 0.001), while the age effect was not (Λ = 0.93, F(3,48) = 1.2, *p* = 0.31). Pairwise tests revealed no significant differences between the positive SZ patients and controls, though a trend of an MPF decrease was observed for GM (Table 2). SZ patients with negative symptoms had significantly lower MPF in all tissues as compared to controls. SZ patients with negative symptoms also showed a trend of an MPF reduction relative to the patients with positive symptoms in all tissues, while the significant difference was observed for WM only (Table 2).

**Table 2:** MPF values (Mean ± SD) in segmented brain tissues of the control and SZ patients groups.

Group	GM MPF (%)	PVWGM MPF (%)	WM MPF (%)
Controls	6.44 ± 0.24	9.22 ± 0.40	13.04 ± 0.56
All SZ patients (*P* vs. controls)	6.12 ± 0.28 (<0.001)	8.88 ± 0.41 (0.004)	12.63 ± 0.63 (0.02)
Positive SZ (*P* vs. controls)	6.21 ± 0.35 (0.07)	9.07 ± 0.43 (0.60)	13.02 ± 0.56 (0.99)
Negative SZ (*P* vs. controls; *P* vs. positive SZ)	6.07 ± 0.24 (<0.001; 0.37)	8.79 ± 0.37 (0.002; 0.17)	12.45 ± 0.58 (0.003; 0.03)

**Fig. 2:**
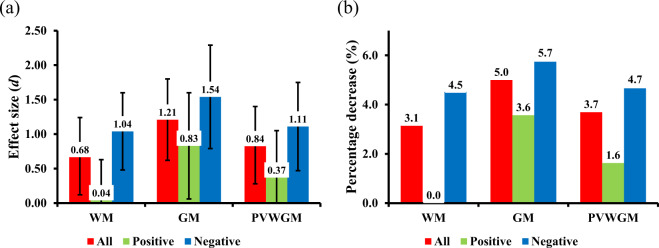
Mean decrease in MPF in segmented brain tissues (WM, GM, PVWGM) of SZ patients relative to the control group. An MPF reduction is expressed as the effect size (Cohen’s d) (**a**) and percentage change (**b**). Colors indicate the data for all SZ patients (red) and subgroups of SZ patients with leading positive (green) and negative (blue) symptoms. Error bars correspond to the 95% confidence intervals for the effect sizes.

Percentage changes in MRI parameters between the subject groups and the corresponding effect sizes (Cohen’s d) are presented in Fig. 2. MPF in GM demonstrated the largest decrease in SZ patients relative to controls as compared to other tissue classes. The percentage changes and effect sizes for MPF in the mixed PVWGM tissue showed intermediate values between those in pure WM and GM. In all tissues, a decrease in MPF was substantially larger for patients with leading negative symptoms as compared to the patients with positive symptoms (Fig. 2).

Correlations between MPF in brain tissues and clinical variables for SZ patients are presented in Table 3. In the control group, MPF significantly negatively correlated with age in GM (*r* = −0.45, *P* = 0.03) and showed a trend of an age-related decrease in PVWGM (*r* = −0.40, *P* = 0.06) and WM (*r* = −0.34, *P* = 0.11). In SZ patients (Table 3), a marginally significant negative correlation between MPF in WM and age was found. Among the clinical variables, MPF significantly negatively correlated with the disease duration only (Table 3), particularly in WM and PVWGM with a stronger relationship (*r* = −0.51, *P* = 0.004) identified for WM. These correlations retained significance after adjustment for age (partial *r* = −0.39, *P* = 0.04 for PVWGM and partial *r* = −0.38, *P* = 0.04 for WM).

**Table 3:** Correlations (Pearson’s r) between MPF in segmented brain tissues and clinical variables in SZ patients.

Clinical variable	GM MPF (%)	PVWGM MPF (%)	WM MPF (%)
Age	−0.19 (*p* = 0.31)	−0.18 (*p* = 0.34)	−**0.37** (***p*** = **0.04**)
Disease duration	−0.33 (*p* = 0.07)	−**0.37** (***p*** = **0.04**)	−**0.51** (***p*** = **0.004**)
Onset age	0.15 (*p* = 0.42)	0.21 (*p* = 0.26)	0.06 (*p* = 0.74)
CPZ equivalent dose	−0.26 (*p* = 0.16)	−0.15 (*p* = 0.41)	−0.12 (*p* = 0.52)

Results of the statistical analysis of scan-rescan repeatability of MPF measurements in brain tissues for a subgroup of control participants are summarized in Table S1, and the corresponding Bland-Altman plot is presented in Fig. S1 (Supplementary Material). Repeated MPF measurements demonstrated high reproducibility with remarkably small CoV (1% for GM, 0.9% for PVWGM, and 0.8% for WM) and the absence of significant biases.

## Discussion

This study provides the first report of quantitative myelin assessment in SZ using the fast MPF mapping method^45,46^. Our results indicate the global myelin loss in both WM and GM of SZ patients, which is associated with the disease duration and dominant-negative symptoms. Our findings are in agreement with the evidence of myelin damage in SZ^8–10,14,15^ and corroborate the results of earlier studies obtained using different myelin-sensitive MRI methods^35,36,38^. Our results also add confidence to the understanding of DTI and MTR abnormalities in SZ^23–26,29^ as at least partially related to myelin, though other pathological factors, such as axonal remodeling and degeneration, inflammation, and iron content changes need to be taken into account in the interpretation of non-specific to myelin imaging measures.

The results of this study suggest that myelin loss in GM and WM of SZ patients may have different pathological mechanisms. Both the percentage MPF changes and effect sizes indicate that the relative reduction in the myelin content is substantially larger for GM as compared to WM. The observed effects are corroborated by the high reproducibility of MPF measurements according to the scan-rescan data presented in the Supplementary material. The distinctions between WM and GM appear even more dramatic in terms of the absolute myelin content changes. The amount of myelin in brain tissues can be estimated from the histologically validated linear relationship between MPF and myelin density according to the formula MPF = 0.21 × MD + 3.9, where MD is the myelin density expressed in percentage units based on histological staining^33^. While the exact coefficients in the above equation may vary depending on the pathological material, MPF mapping technique, and histology quantitation approach, this transformation provides an advantage of eliminating the non-myelin contribution (or a substantial portion of it) from MPF, thus resulting in a more realistic representation of the relative myelin content in WM and GM^33^. A similar approach was recently used to approximate myelin content changes in WM and GM during adolescent brain development^51^. Application of this estimation technique to the data from Table 2 results in the myelin densities in GM and WM of SZ patients of 10.6% and 41.6% and the corresponding values for controls of 12.1% and 43.5%, respectively. Accordingly, the relative myelin loss in SZ in GM is approximately 13% in contrast to only an about 5% reduction in WM. Despite this large difference, WM myelination showed a much stronger negative correlation with the disease duration as compared to GM. We believe that such distinctions may be indicative of different temporal trajectories of GM and WM dysmyelination in SZ. Particularly, the myelin deficiency in GM may represent either a pre-existing trait associated with the developmental SZ etiology or a result of rapid progression in the early disease stage. On the other hand, myelin loss in WM could be described by a slow progressive pattern associated with such pathological processes as neurodegeneration, inflammatory demyelination, and excitotoxic damage to oligodendrocytes^1–7^. These findings suggest an intriguing hypothesis of SZ as a two-stage disease in terms of myelin damage, where GM dysmyelination reflects the disrupted intracortical connectivity in the early disease manifestation and follows by the gradual breakdown of long-range connectivity associated with WM demyelination in chronic disease.

It is known that negative symptoms of SZ have an adverse impact on psychosocial functioning and disease outcome and show less response to antipsychotic therapy. Our results indicate that patients with leading negative symptoms have a more pronounced decrease in myelin density as compared to the patients with positive symptoms. The results of this study are consistent with the literature data on a decrease in the volume of white and gray matter in negative SZ. The meta-analysis by the ENIGMA-Schizophrenia consortium indicates an association between cortical thinning in prefrontal regions and the severity of negative symptoms in SZ^63^. In smaller samples of patients, an inverse correlation was found between the volume of white matter and negative symptoms of SZ^64^. The voxel-wise analysis demonstrated significant fractional anisotropy reductions in the WM tracts in chronic negative SZ which indicates more pronounced microstructural changes^65^. Proteomic analysis of the area-specific for cognitive impairments in SZ revealed dysfunction of pathways associated with the regulation of synaptic processes in the caudate nucleus, cerebellum, and cingulate gyrus^66^. Associations of leading negative symptoms and impairment in the prefrontal cortex, hippocampus^67^, amygdala^68^, as well as a metabolic disturbance in the prefrontal-thalamic-parietal network^63^ were found. As such, more extensive myelin damage in SZ with negative symptoms appears a biologically plausible finding, which is in line with a general view of the greater severity of this disease phenotype and its associations with anatomical and functional brain abnormalities.

Correlation analysis revealed significant negative correlations between the disease duration and MPF in WM and PVWGM, which remained significant after adjustment for age. As such, a decrease in myelin density in chronic SZ patients cannot be explained by changes in myelination associated with normal aging^69,70^. At the same time, negative correlations between MPF and age in healthy controls were found, being in agreement with the age-related trends for the adult population^69,70^. Such an age effect seems to be suppressed by disease-related factors in SZ, as indicated by weaker correlations between MPF and age found in patients. The loss of myelin-associated with the disease duration is in line with the disease-related brain atrophy^22^ and a decrease of WM integrity^71^ reported in earlier SZ studies.

*This study has several limitations*. First, the group of patients with leading negative symptoms had a longer duration of the disease as compared to the group with positive symptoms. However, the fact that negative symptoms predominate in long-term illness is well known^72^. As such, it is difficult or even impossible to avoid this confounder within the study design with consecutive patient recruitment. Second, a relatively small number of patients with positive symptoms was recruited for this study. Such patients frequently present with acute psychosis, which makes it difficult to achieve a sufficient level of cooperation for participation in an imaging research study. Third, the cross-sectional design of this pilot study limits the mechanistic interpretation of inferred temporal changes, particularly in relationships to such factors as age and disease duration. While our results indicate substantial disconnection between WM and GM myelination in SZ, this construal should be taken with caution and tested in future longitudinal studies. Fourth, a possible effect of antipsychotic therapy on the myelin content cannot be ruled out, although we did not find significant correlations with the antipsychotic dose. The question about the effect of antipsychotics on brain morphology and microstructure remains controversial^73,74^. Future studies with the inclusion of drug-naïve patients are warranted to address this issue. Finally, our interpretation of an MPF decreases solely in terms of myelin loss may be oversimplified, as MPF could be also affected to some extent by the tissue water content^53^. An increased water content theoretically may be related to inflammation, which is considered one of the possible mechanisms of microstructural tissue damage in the SZ brain^5,75^. However, an earlier study^53^ did not find correlations between MPF and post-ischemic microglial activation in an animal stroke model, suggesting that myelin remains the main determinant of MPF in brain tissues even in the presence of severe inflammation and edema. Furthermore, oligodendroglial injury, demyelination, and neuroinflammation are commonly viewed as interrelated phenomena in SZ and other brain diseases^5^. A recent modification of the MPF mapping technique enabling correction of MPF values for water content changes^76,77^ may be helpful to investigate this aspect of SZ pathology in more detail in the future.

*In conclusion*, this study demonstrated that chronic SZ is characterized by global microscopic brain hypomyelination of both WM and GM, which can be detected and quantified by the fast MPF mapping method. A decrease in the myelin content in the SZ brain is associated with the disease duration and negative symptoms, thus suggesting the clinical relevance of myelin damage in this disease. Our results underscore the importance of myelin pathology for understanding the biological mechanisms of SZ development and substantiate the interpretation of earlier microstructural neuroimaging findings in terms of myelin deficiency. In the methodological aspect, this study provides technical background for prospective applications of the fast MPF mapping method in clinical psychiatric research.

## Supplementary information

Supplementary Material

## References

[1] Davis KL (2003). White matter changes in schizophrenia: evidence for myelin-related dysfunction. Arch. Gen. Psychiatry.

[2] Takahashi N, Sakurai T, Davis KL, Buxbaum JD (2011). Linking oligodendrocyte and myelin dysfunction to neurocircuitry abnormalities in schizophrenia. Prog. Neurobiol..

[3] Maas, D. A., Vallès, A., Martens, G. J. M. Oxidative stress, prefrontal cortex hypomyelination and cognitive symptoms in schizophrenia. *Transl. Psychiatry*10.1038/tp.2017.138 (2017). 10.1038/tp.2017.138 PMC5538118 28934193

[4] Mighdoll MI, Tao R, Kleinman JE, Hyde TM (2015). Myelin, myelin-related disorders, and psychosis. Schizophr. Res..

[5] Bernstein HG, Steiner J, Guest PC, Dobrowolny H, Bogerts B (2015). Glial cells as key players in schizophrenia pathology: recent insights and concepts of therapy. Schizophr. Res..

[6] Gouvêa-Junqueira, D. et al. Novel treatment strategies targeting myelin and oligodendrocyte dysfunction in schizophrenia. *Front. Psychiatry*10.3389/fpsyt.2020.00379 (2020). 10.3389/fpsyt.2020.00379 PMC7203658 32425837

[7] Raabe, F. J. et al. Oligodendrocytes as a new therapeutic target in schizophrenia: from histopathological findings to neuron-oligodendrocyte interaction. *Cells*10.3390/cells8121496 (2019). 10.3390/cells8121496 PMC6952785 31771166

[8] Miyakawa T (1972). Electron microscopic study on schizophrenia. Acta Neuropathol..

[9] Uranova NA, Vikhreva OV, Rachmanova VI, Orlovskaya DD (2011). Ultrastructural alterations of myelinated fibers and oligodendrocytes in the prefrontal cortex in schizophrenia: a postmortem morphometric study. Schizophr. Res. Treat..

[10] Vikhreva OV, Rakhmanova VI, Orlovskaya DD, Uranova NA (2016). Ultrastructural alterations of oligodendrocytes in prefrontal white matter in schizophrenia: a post-mortem morphometric study. Schizophr. Res..

[11] Martins-de-Souza D (2010). Proteome and transcriptome analysis suggests oligodendrocyte dysfunction in schizophrenia. J. Psychiatr. Res..

[12] Hakak Y (2001). Genome-wide expression analysis reveals dysregulation of myelination-related genes in chronic schizophrenia. Proc. Natl Acad. Sci. USA.

[13] Tkachev D (2003). Oligodendrocyte dysfunction in schizophrenia and bipolar disorder. Lancet.

[14] Parlapani E (2009). Association between myelin basic protein expression and left entorhinal cortex pre-alpha cell layer disorganization in schizophrenia. Brain Res..

[15] Lake EMR (2017). Altered intracortical myelin staining in the dorsolateral prefrontal cortex in severe mental illness. Eur. Arch. Psychiatry Clin. Neurosci..

[16] Schmitt A (2009). Stereologic investigation of the posterior part of the hippocampus in schizophrenia. Acta Neuropathol..

[17] Falkai P (2016). Decreased oligodendrocyte and neuron number in anterior hippocampal areas and the entire hippocampus in schizophrenia: a stereological postmortem study. Schizophr. Bull..

[18] Uranova NA, Vostrikov VM, Orlovskaya DD, Rachmanova VI (2004). Oligodendroglial density in the prefrontal cortex in schizophrenia and mood disorders: a study from the Stanley Neuropathology Consortium. Schizophr. Res..

[19] Hof PR (2003). Loss and altered spatial distribution of oligodendrocytes in the superior frontal gyrus in schizophrenia. Biol. Psychiatry.

[20] Mauney SA, Pietersen CY, Sonntag KC, Woo TU (2015). Differentiation of oligodendrocyte precursors is impaired in the prefrontal cortex in schizophrenia. Schizophr. Res..

[21] Shenton ME, Dickey CC, Frumin M, McCarley RW (2001). A review of MRI findings in schizophrenia. Schizophr. Res..

[22] Olabi B (2011). Are there progressive brain changes in schizophrenia? A meta-analysis of structural magnetic resonance imaging studies. Biol. Psychiatry.

[23] Kubicki M, Shenton ME (2014). Diffusion tensor imaging findings and their implications in schizophrenia. Curr. Opin. Psychiatry.

[24] Karlsgodt KH (2016). Diffusion imaging of white matter in schizophrenia: progress and future directions. Biol. Psychiatry Cogn. Neurosci. Neuroimaging.

[25] Kelly S (2018). Widespread white matter microstructural differences in schizophrenia across 4322 individuals: results from the ENIGMA Schizophrenia DTI Working Group. Mol. Psychiatry.

[26] Scheel M, Prokscha T, Bayerl M, Gallinat J, Montag C (2013). Myelination deficits in schizophrenia: evidence from diffusion tensor imaging. Brain Struct. Funct..

[27] Wheeler-Kingshott CA, Cercignani M (2009). About “axial” and “radial” diffusivities. Magn. Reson. Med..

[28] Underhill HR, Yuan C, Yarnykh VL (2009). Direct quantitative comparison between cross-relaxation imaging and diffusion tensor imaging of the human brain at 3.0 T. Neuroimage.

[29] Palaniyappan L, Al-Radaideh A, Mougin O, Gowland P, Liddle PF (2013). Combined white matter imaging suggests myelination defects in visual processing regions in schizophrenia. Neuropsychopharmacology.

[30] Antosik-Biernacka A (2006). Magnetization transfer imaging in chronic schizophrenia. Med. Sci. Monit..

[31] Mandl RCW (2010). Tract-based analysis of magnetization transfer ratio and diffusion tensor imaging of the frontal and frontotemporal connections in schizophrenia. Schizophr. Bull..

[32] de Weijer AD (2011). Microstructural alterations of the arcuate fasciculus in schizophrenia patients with frequent auditory verbal hallucinations. Schizophr. Res..

[33] Underhill HR, Rostomily RC, Mikheev AM, Yuan C, Yarnykh VL (2011). Fast bound pool fraction imaging of the in vivo rat brain: Association with myelin content and validation in the C6 glioma model. Neuroimage.

[34] Yarnykh VL (2015). Fast whole-brain three-dimensional macromolecular proton fraction mapping in multiple sclerosis. Radiology.

[35] Flynn SW (2003). Abnormalities of myelination in schizophrenia detected in vivo with MRI, and post-mortem with analysis of oligodendrocyte proteins. Mol. Psychiatry.

[36] Vanes LD, Mouchlianitis E, Wood TC, Shergill SS (2018). White matter changes in treatment refractory schizophrenia: does cognitive control and myelination matter?. Neuroimage Clin..

[37] Lang DJ (2014). 48 echo T_2_ myelin imaging of white matter in first-episode schizophrenia: evidence for aberrant myelination. Neuroimage Clin..

[38] Iwatani, J. et al. Use of T1-weighted/T2-weighted magnetic resonance ratio images to elucidate changes in the schizophrenic brain. *Brain Behav*. 10.1002/brb3.399 (2015). 10.1002/brb3.399 PMC4614056 26516617

[39] Wei W (2020). Depth-dependent abnormal cortical myelination in first-episode treatment-naïve schizophrenia. Hum. Brain. Mapp..

[40] Birkl C (2019). The influence of brain iron on myelin water imaging. Neuroimage.

[41] Uddin, M. N., Figley, T. D., Marrie, R. A., Figley, C. R. Can T1w/T2w ratio be used as a myelin-specific measure in subcortical structures? Comparisons between FSE-based T1w/T2w ratios, GRASE-based T1w/T2w ratios and multi-echo GRASE-based myelin water fractions. *NMR Biomed*. 10.1002/nbm.3868 (2018). 10.1002/nbm.3868 29315894

[42] Sadrzadeh SM, Saffari Y (2004). Iron and brain disorders. Am. J. Clin. Pathol..

[43] Helms G, Dathe H, Kallenberg K, Dechent P (2008). High-resolution maps of magnetization transfer with inherent correction for RF inhomogeneity and T1 relaxation obtained from 3D FLASH MRI. Magn. Reson. Med..

[44] Varma G, Duhamel G, de Bazelaire C, Alsop DC (2015). Magnetization transfer from inhomogeneously broadened lines: a potential marker for myelin. Magn. Reson. Med..

[45] Yarnykh VL (2012). Fast macromolecular proton fraction mapping from a single off-resonance magnetization transfer measurement. Magn. Reson. Med..

[46] Yarnykh VL (2016). Time-efficient, high-resolution, whole brain three-dimensional macromolecular proton fraction mapping. Magn. Reson. Med..

[47] Yarnykh VL (2018). Iron-insensitive quantitative assessment of subcortical gray matter demyelination in multiple sclerosis using the macromolecular proton fraction. Am. J. Neuroradiol..

[48] Petrie EC (2014). Neuroimaging, behavioral, and psychological sequelae of repetitive combined blast/impact mild traumatic brain injury in Iraq and Afghanistan War Veterans. J. Neurotrauma.

[49] Yarnykh VL, Prihod’ko IY, Savelov AA, Korostyshevskaya AM (2018). Quantitative assessment of normal fetal brain myelination using fast macromolecular proton fraction mapping. Am. J. Neuroradiol..

[50] Korostyshevskaya AM, Prihod’ko IY, Savelov AA, Yarnykh VL (2019). Direct comparison between apparent diffusion coefficient and macromolecular proton fraction as quantitative biomarkers of the human fetal brain maturation. J. Magn. Reson. Imaging.

[51] Corrigan, N. M. et al. Myelin development in cerebral gray and white matter during adolescence and late childhood. *Neuroimage*10.1016/j.neuroimage.2020.117678 (2021). 10.1016/j.neuroimage.2020.117678 PMC8214999 33359342

[52] Khodanovich, M. Y. et al. Histological validation of fast macromolecular proton fraction mapping as a quantitative myelin imaging method in the cuprizone demyelination model. *Sci. Rep*. 10.1038/srep46686 (2017). 10.1038/srep46686 PMC5402392 28436460

[53] Khodanovich MY (2018). Quantitative assessment of demyelination in ischemic stroke in vivo using macromolecular proton fraction mapping. J. Cereb. Blood Flow. Metab..

[54] Khodanovich, M. et al. Quantitative imaging of white and gray matter remyelination in the cuprizone demyelination model using the macromolecular proton fraction. *Cells*10.3390/cells8101204 (2019). 10.3390/cells8101204 PMC6830095 31590363

[55] Mancini, M. et al. An interactive meta-analysis of MRI biomarkers of myelin. *Elife*10.7554/eLife.61523 (2020). 10.7554/eLife.61523 PMC7647401 33084576

[56] Yarnykh VL, Kisel AA, Khodanovich MY (2020). Scan-Rescan Repeatability and Impact of B(0) and B(1) Field Nonuniformity Corrections in Single-Point Whole-Brain Macromolecular Proton Fraction Mapping. J. Magn. Reson. Imaging.

[57] Naumova AV, Akulov AE, Khodanovich MY, Yarnykh VL (2017). High-resolution three-dimensional macromolecular proton fraction mapping for quantitative neuroanatomical imaging of the rodent brain in ultra-high magnetic fields. Neuroimage.

[58] Anisimov NV, Pavlova OS, Pirogov YA, Yarnykh VL (2020). Three-dimensional fast single-point macromolecular proton fraction mapping of the human brain at 0.5 Tesla. Quant. Imaging Med. Surg..

[59] World Health Organization (WHO). The ICD-10 Classification of Mental and Behavioural Disorders. (World Health Organization, Genève, Switzerland, 1993).

[60] Yarnykh, V. & Korostyshevskaya, A. Implementation of fast macromolecular proton fraction mapping on 1.5 and 3 Tesla clinical MRI scanners: preliminary experience. *J. Phys*. 10.1088/1742-6596/886/1/012010 (2017).

[61] Smith SM (2002). Fast robust automated brain extraction. Hum. Brain Mapp..

[62] Zhang Y, Brady M, Smith S (2001). Segmentation of brain MR images through a hidden Markov random field model and the expectation-maximization algorithm. IEEE Trans. Med. Imaging.

[63] Walton E (2018). Prefrontal cortical thinning links to negative symptoms in schizophrenia via the ENIGMA consortium. Psychol. Med..

[64] Galderisi S, Merlotti E, Mucci A (2015). Neurobiological background of negative symptoms. Eur. Arch. Psychiatry Clin. Neurosci..

[65] Asami T (2014). Cerebral white matter abnormalities and their associations with negative but not positive symptoms of schizophrenia. Psychiatry Res..

[66] Reis-de-Oliveira, G. et al. Digging deeper in the proteome of different regions from schizophrenia brains. *J. Proteomics*10.1016/j.jprot.2020.103814 (2020). 10.1016/j.jprot.2020.103814 32389842

[67] Howes, O. D., McCutcheon. R. J., Owen, M. & Murray, R. M. The role of genes, stress, and dopamine in the development of schizophrenia. *Biol. Psychiatry* **81**, 9–20 (2017). 10.1016/j.biopsych.2016.07.014 PMC5675052 27720198

[68] Rahm C (2015). Negative symptoms in schizophrenia show association with amygdala volumes and neural activation during affective processing. Acta Neuropsychiatr..

[69] Kochunov P (2011). Fractional anisotropy of cerebral white matter and thickness of cortical gray matter across the lifespan. Neuroimage.

[70] Grydeland H, Walhovd KB, Tamnes CK, Westlye LT, Fjell AM (2013). Intracortical myelin links with performance variability across the human lifespan: results from T1- and T2-weighted MRI myelin mapping and diffusion tensor imaging. J. Neurosci..

[71] Mori T (2007). Progressive changes of white matter integrity in schizophrenia revealed by diffusion tensor imaging. Psychiatry Res..

[72] Fenton WS, McGlashan TH (1991). Natural history of schizophrenia subtypes. II. Positive and negative symptoms and long-term course. Arch. Gen. Psychiatry.

[73] Navari S, Dazzan P (2009). Do antipsychotic drugs affect brain structure? A systematic and critical review of MRI findings. Psychol. Med..

[74] Huhtaniska, S. et al. Long-term antipsychotic use and brain changes in schizophrenia-a systematic review and meta-analysis. *Hum. Psychopharmacol*. 10.1002/hup.2574 (2017).. 10.1002/hup.2574 28370309

[75] Müller N (2018). Inflammation in schizophrenia: pathogenetic aspects and therapeutic considerations. Schizophr. Bull..

[76] Mossahebi P, Alexander AL, Field AS, Samsonov AA (2015). Removal of cerebrospinal fluid partial volume effects in quantitative magnetization transfer imaging using a three-pool model with nonexchanging water component. Magn. Reson. Med..

[77] Mossahebi P, Yarnykh VL, Samsonov A (2014). Analysis and correction of biases in cross-relaxation MRI due to biexponential longitudinal relaxation. Magn. Reson. Med..

