# Supplementary material for: Global hypomyelination of the brain white and gray matter in schizophrenia: quantitative imaging using macromolecular proton fraction
Source: Transl Psychiatry. 2021 Jun 30;11:365. doi: 10.1038/s41398-021-01475-8 (PMC8257619; doi:10.1038/s41398-021-01475-8)
Supplement: Supplementary file 1 — Supplementary Material [file 41398_2021_1475_MOESM1_ESM.docx]

**Global hypomyelination of the brain white and gray matter in schizophrenia: quantitative imaging using macromolecular proton fraction**

Liudmila P. Smirnova, [Vasily L. Yarnykh](https://www.ncbi.nlm.nih.gov/pubmed/?term=Yarnykh%20VL%5BAuthor%5D&cauthor=true&cauthor_uid=25208343), Daria A. Parshukova, Elena G. Kornetova, Arkadiy V. Semke, Anna V. Usova, Anna O. Pishchelko, Marina Y. Khodanovich, and Svetlana A. Ivanova

**Supplementary Material**

**Table S1** Scan-rescan repeatability of MPF measurements in segmented brain tissues.

| Value | GM | PVWGM | WM |
| --- | --- | --- | --- |
| Mean MPF ± SD, 1^st^ scan (%) | 6.34 ± 0.21 | 9.36 ± 0.34 | 13.43 ± 0.34 |
| Mean MPF ± SD, 2^nd^ scan (%) | 6.30 ± 0.17 | 9.30 ± 0.30 | 13.39 ± 0.29 |
| Mean difference ± SD (%) | 0.03 ± 0.09 | 0.06 ± 0.12 | 0.04 ± 0.16 |
| 95% Limits of agreement (%) | -0.15; 0.22 | -0.17; 0.28 | -0.28; 0.36 |
| Significance of bias (p) | 0.32 | 0.19 | 0.54 |
| Within-subject CoV (%) | 1.0 | 0.9 | 0.8 |


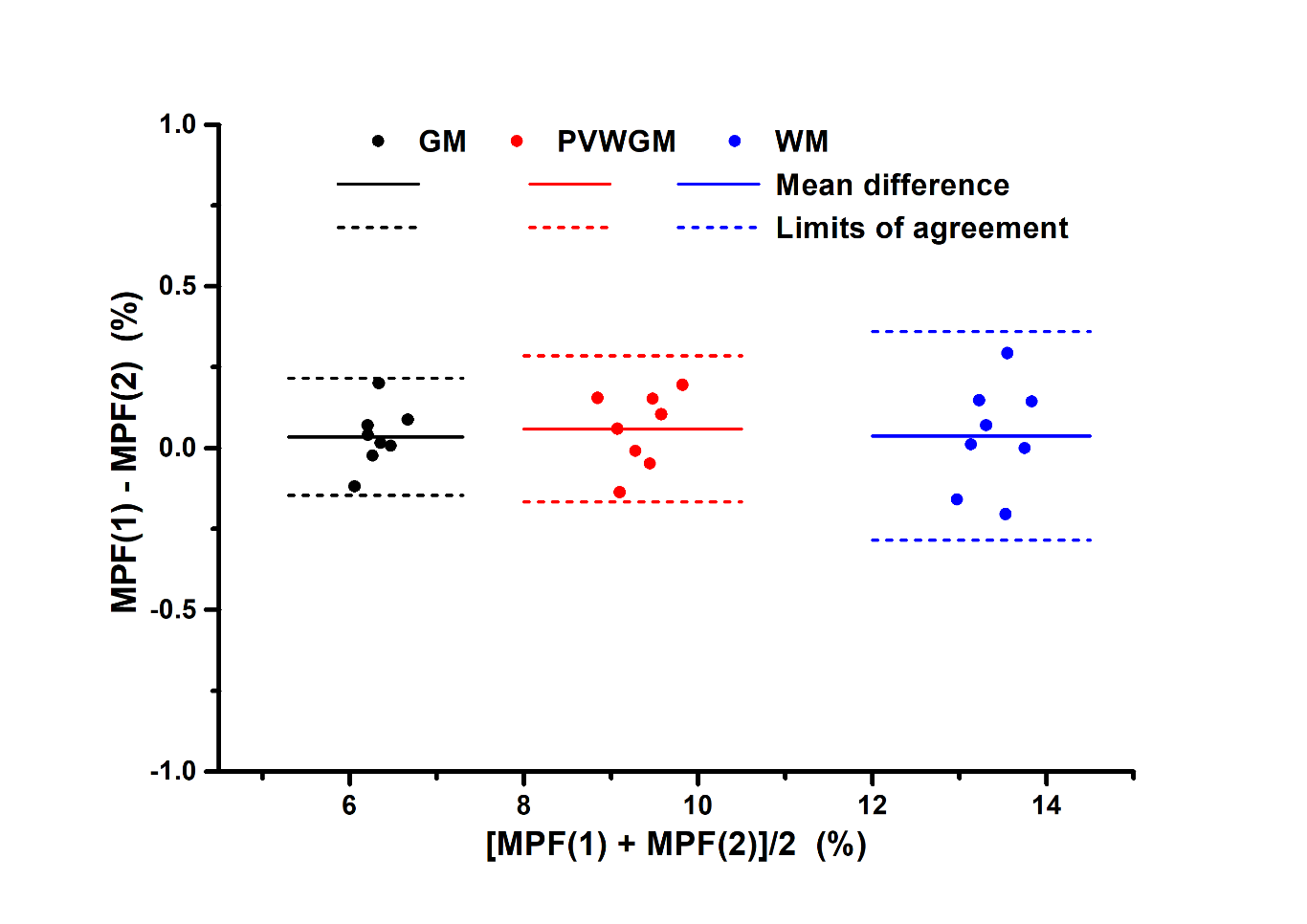


**Fig. S1** Bland-Altman plot for MPF measurements in segmented brain tissues obtained from two repeated scans in eight healthy control subjects.
